# Improved Inference of Gene Regulatory Networks through Integrated Bayesian Clustering and Dynamic Modeling of Time-Course Expression Data

**DOI:** 10.1371/journal.pone.0068358

**Published:** 2013-07-23

**Authors:** Brian Godsey

**Affiliations:** Department of Statistics and Probability Theory, Vienna University of Technology, Vienna, Austria; CRS4, Italy

## Abstract

Inferring gene regulatory networks from expression data is difficult, but it is common and often useful. Most network problems are under-determined–there are more parameters than data points–and therefore data or parameter set reduction is often necessary. Correlation between variables in the model also contributes to confound network coefficient inference. In this paper, we present an algorithm that uses integrated, probabilistic clustering to ease the problems of under-determination and correlated variables within a fully Bayesian framework. Specifically, ours is a dynamic Bayesian network with integrated Gaussian mixture clustering, which we fit using variational Bayesian methods. We show, using public, simulated time-course data sets from the *DREAM4 Challenge*, that our algorithm outperforms non-clustering methods in many cases (7 out of 25) with fewer samples, rarely underperforming (1 out of 25), and often selects a non-clustering model if it better describes the data. Source code (*GNU Octave*) for BAyesian Clustering Over Networks (*BACON*) and sample data are available at: http://code.google.com/p/bacon-for-genetic-networks.

## Introduction

Inferring gene regulatory networks from high-throughput gene expression data is a difficult task, in particular because of the high number of genes relative to the number of data points, and also because of the random noise that is present in measurement. Over the last several years, many new methods have been developed to address this problem; a nice review of these can be found in [1]. This review directly compares several different types of approaches by summarizing the correctness of the genetic networks inferred from synthetic (*in silico*) data generated from a known network. Of particular interest are the results of each of the algorithms when applied to the *DREAM4 In Silico Network Challenge* data sets, which includes data types such as “knock-out”, “knock-down”, and time-series data among the sub-challenges. See [2] for more details on the *DREAM* challenges.

Though [3] have had success combining methods in order to infer genetic networks from different types of data simultaneously, here we focus on time-series data and the corresponding methods for network inference. In the review of [7], two types of algorithms seem to outperform the others when applied to time-series data: dynamic Bayesian networks and causal structure identification (CSI) in non-linear dynamical systems (NDSs).

Dynamic Bayesian networks (DBNs) are typically some variation of the basic linear model
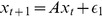
(1)

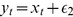
(2)where in the context of gene regulatory networks, 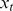 is the vector of “true” gene expression levels at time 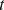, 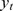 is a vector of observations of these expression levels, 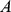 is a matrix of interaction coefficients, and 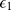 and 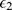 are random (Gaussian) noise. More information on DBNs and their application to gene regulatory networks can be found in [4] and [5].

The algorithms considered in [1] include a model very similar to that of the basic DBN formulation above, but which exploits conditional [first-order] dependence within nodes of the network, as well as an assumption of relative sparseness, to efficiently infer network structure. This model, from [6] is referred to as *G1DBN* and is available as an *R* package from *CRAN* [7]. The second DBN considered by [1] is that of [8], which adapts a state-space model with inputs to include hidden states, the quantity and values of which are inferred through variational Bayesian learning. This algorithm is referred to as *VBSSM*, as in the review. Causal structure identification (CSI) in non-linear dynamical systems (NDSs) avoids the restriction of linearity when determining network structure, and in the case of [3], which is also considered in the review, latent interaction parameters of a discrete Gaussian process model are inferred using a Bayesian framework. According to [1], both the *G1DBN* and *VBSSM* algorithms performed well on the *DREAM4* data sets, as did the CSI algorithm of [9]. Both DBNs and CSI outperformed ordinary differential equations (ODEs) and models using Granger causality.

Though these results are convincing, there is still room for improvement, and the discussion of optimal methods is still open; in fact, the body of research in the area of gene expression time-series analysis continues to grow quickly. A recent review, [10], outlines the state of the art in gene expression time-series analysis, including much information on clustering methods and software. We can see that, when compared to a similar, earlier review, [11], a considerable amount of work has been done. However, we feel that there is still a branch of time-series data analysis that is under-utilized in gene regulatory network inference. Despite the vast amount of work that has been done on the clustering of gene expression data, much of which deals specifically with time-series, relatively little work has been done on inferring time-dependent interactions between gene clusters or between a gene cluster and an individual gene. Let us briefly discuss clustering methods for time-series data before continuing on to its potential use in inferring gene regulatory networks.

In order to successfully cluster time-series data, we need to utilize the stronger dependencies between data in consecutive time points relative to more distant time points. Quite often, researchers are interested in expression patterns across time; [12] cluster short time-series data around specific pre-determined profiles that may have meaning within the particular experiment. [13] perform a similar cluster analysis of time-series, but instead of using pre-determined expression patterns, they use a hidden Markov model (HMM) to infer dynamics of a limited number of clusters between a small number of states (*e.g.* nine discrete expression levels). [14] take a slightly different approach by clustering genes to inferred profiles, focusing mainly on impulse models in experiments where one might expect peaks in the expression values.

In each of the above papers, it was shown that gene clustering can infer biological meaning, whether co-expression, co-regulation, involvement in particular biological processes, or some other effect. Such information may also be valuable in inferring genetic regulatory networks. [15] and [16] have done work in this direction, combining state-space models and clustering heuristics for simultaneous, integrated inference. However, both of these are demonstrated on data containing hundreds of genes which are clustered or grouped into a low (fewer than 20) number of clusters/modules and subsequently the large cluster size prevents any meaningful conclusions about regulatory interactions between specific genes.

In this paper, we describe a fully Bayesian model of gene cluster interaction, and we demonstrate that probabilistic gene clustering in conjunction with a dynamic Bayesian network can aid in the inference of gene regulatory networks, even in the *DREAM4* data sets, where no clusters were explicitly included. It achieves this by potentially reducing–in a fully Bayesian manner–the parameter space and helping solve the problem of solution identifiability in under-defined, noisy data models such as are common in gene expression analysis. The algorithm presented here is a variational Bayesian hybrid of a DBN and a Gaussian mixture clustering algorithm, both of which have been shown to infer meaningful solutions to their respective problems [8], [17], and which we show can work even better in tandem. We call this algorithm *BAyesian Clustering Over Networks* (*BACON*). *BACON* is built specifically to simultaneously consider multiple data sets based on the same network, such that for each data set, expression states are inferred independently, but that cluster membership and regulatory dynamics are assumed to be constant for all data from the given network, regardless of the particular data set. This gives more accurate results than a heuristic combination of interaction rankings based on the various time-series for each of the *DREAM4* networks.

## Methods

In this paper we introduce an algorithm called *BACON*, which is a variational Bayesian algorithm that combines a Gaussian mixture clustering model with a DBN. However, before we give the specific formulation of our model, it may be helpful first to look at a simple case where integrated clustering can help infer gene regulatory networks, even if no “true” clusters are present.

### A simple example

Assume, as an illustration, that we have a three genes, X, Y, and Z and that we have time-series expression data for each of them, such that the observed expression levels of these at time 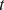 are given by 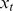, 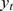, and 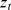, respectively, for time points 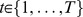. Let us, for simplicity's sake, assume that we are concerned only with potenial regulators of gene Z, and that X and Y are the only two candidates. Furthermore, we assume a simple linear model of dynamics, in which 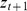 is assumed to be a noisy observation of the dot/inner product of the vector of two interaction coefficients 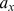 and 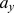 with the vector of 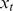 and 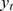, namely:
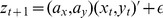
(3)


Note that this is a simple linear model on three variables, where all interaction coefficients except 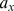 and 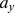 are set to zero. It is a special case of the standard linear model, given in equation 1, where for this example we treat the observations as the true expression values, we attempt only to infer 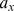 and 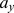, and for illustration purposes we ignore all other possible interaction coefficients. When attempting to infer 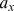 and 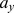 under a Bayesian framework, we make the following assumptions:
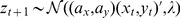
(4)

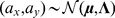
(5)where 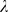 is a precision parameter (inverse of variance), 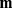 is the prior mean of the multivariate normal distribution, and 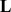 is a 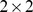 precision matrix (inverse of the covariance matrix).

Given these assumptions, the data 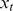, 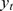, and 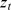, and the precision parameter 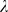 (fixed), the estimated posterior distribution for 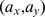 under a variational Bayesian framework (see [?] and [?] for a detailed explanation) is multivariate normal, with mean 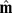 and precision 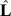, such that
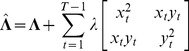
(6)

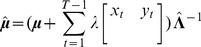
(7)


Under some conditions, such inference works quite well, but if the expression profiles for X and Y are highly correlated (or negatively correlated), then the determinant of 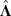 approaches zero, and the diagonal elements of 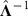 (the estimated variances of 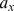 and 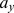) approach infinity. Such a problem can be overcome with a strong prior for 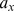 and 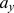, but this is usually not desireable since typically 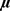 is set to zero (as in [?]), and a high prior precision 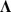 merely pulls the estimate 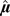 towards zero, and potentially decreases the statistical significance of the inferred interaction parameters. Thus, we are faced with a decision between strong priors or very high variances of posterior parameter estimates.

If the 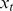 and 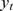 are highly correlated, and if they are likewise correlated with 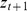, then we might be able to say with near certainty that either X or Y regulates Z, but we could not say which one. This may be acceptable on a small scale, but would be difficult in a gene expression time-series experiment with hundreds of genes and thousands of putative interaction coefficients and covariances. It could be interesting to optimize the choice of a set of, for example, ten gene interactions, with respect to the probability of at least one of them being verifiable in an independent evaluation. But, this would be difficult for experiments with large numbers of genes, and so typically only the individual variances are considered when calculating the statistical significance of the estimated interaction parameter values. Estimating, potentially, thousands of interaction parameters is very difficult in dynamic gene expression time-series analysis because, for example in the basic linear model given in equation 1, there are generally many possible values for the transition matrix, each of which could produce the data. In other words, a given gene expression time-series could be reproduced by many different linear combinations of other [lagged] time-series. A particular case of this is when two potentially regulating genes have highly correlated expression profiles, which, as we have shown above, can cause some difficulty in inference.

Here, we propose that clustering genes and inferring the dynamics of the clusters can help avoid the case in which highly correlated gene profiles inhibit interaction inference. In our example, if genes X and Y have highly correlated expression profiles, then for weak priors the precision estimate in equation 6 is nearly singular, and thus by treating X and Y as two contributors to the same dynamic quantity, we avoid this particular singularity problem altogether. Then, a standard method (DBN or similar) could more easily infer that both X and Y (as one cluster) are likely regulators of Z. If, when creating a list of the most likely individual gene-gene interactions, we simply assign all the inferred interaction coefficients for a cluster to each of its members, we can obtain a ranking of interaction pairs that is comparable to the ranking obtained from a standard DBN.

It may seem, at first, that passing along inferred interaction coefficients to all cluster members would create many false positives. However, if clusters include–by definition–highly correlated expression profiles, then if a cluster appears to be a good potential regulator of a gene, all of the cluster's members must also have profiles that generally indicate potential regulation, and in the absence of clustering, it would be difficult to identify the best interaction parameters. This is true whether or not any or all of the concerned genes are actually verifiable regulators, and thus clustering together correlated expression profiles–regardless of the biological meaning of the clustered genes–could improve inference. For instance, in our example, the presence of gene Y (if highly correlated with X) adversely affects the identification of X as a regulator of Z, a problem that can be avoided if X and Y are treated as members of the same cluster. In a data set with hundreds of genes, the chance of having at least one pair of highly correlated expression profiles is rather large. Of course, we must be careful in our construction of clusters and their dynamics, but as we show, Bayesian inference provides the means to select a number of clusters, to assign cluster membership, and to estimate cluster interaction parameters in an optimal way. We describe this below.

### Model

Given 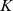 clusters and 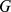 genes, we assume that the cluster expressions 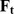 for time points 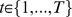 follow the standard linear dynamics
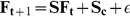
(8)where 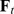 is a vector of length 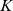, 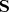 is a 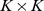 transition matrix, 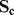 is a column vector of length 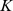, and 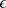 is vector of Gaussian noise. The 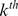 element of 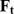, 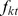, is the expression of cluster 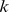 at time 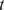. The vector 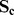 represents linear trends in cluster expression levels over the time points, and its inclusion in the model prevents such trends from being confused for interactions in similarly trending clusters.

The expression of gene 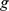 at time 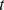 is given by 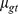, and the membership of gene 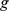 to cluster 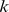 is given by the 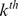 element of the indicator vector 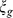. Each gene 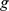 belongs to exactly one cluster 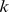, and so 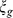 contains a single 1 in in the 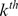 element and zeros elsewhere. The 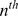 observation/replicate of 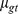 is 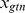. The corresponding prior distributions are:
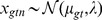
(9)

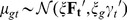
(10)

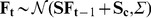
(11)where the 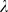 is a technical precision (inverse of variance) representing the measurement errors, assumed to be independent, 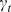 is a vector of precisions of length 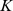, and 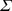 is a 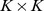 precision matrix which we require to be diagonal, as in [8], so that in the posterior distribution estimates, the rows of 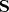 are independent. We also formulate our other prior distributions as in [8]: for the elements of 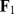, 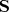, and 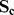, we use zero-mean normal distribution priors whose precisions we iteratively update to maximize the marginal likelihood estimate (discussed below). Likewise, for the hyper-parameters of the gamma distribution priors we assume for the elements of the precisions 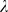, 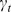, and 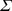. For 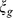, we use a uniform prior distribution over the 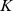 possible clusters.

For multiple time-course data sets from the same gene regulatory network, as we have in the *DREAM4 Challenge* data sets we use in this paper, we infer all of the parameters separately for each of the series, except for the dynamics parameters 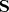 and 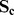 and the membership indicator vectors 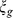, which are shared and inferred simultaneously for all time-series from the given network.

### Inference

To estimate the parameters of our model, we use a variational Bayesian algorithm analagous to those described in [18] and [19], which has been previously used to fit a DBN to gene expression time-series in [8], as well as a Gaussian mixture model for gene clustering in [17].

In short, the algorithm used in this paper estimates the posterior parameter distribution 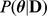 given the data 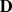 using a factorable distribution 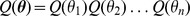 whose factors can be iteratively updated so that with each update, 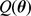 becomes a better approximation for 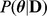, as measured by the Kullback-Leibler divergence between the two. We have chosen conjugate prior distributions for each of the parameters we estimate, and therefore the posterior distribution estimate 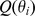 for each parameter is of the same form as its prior, and the parameters of these distributions are updated iteratively according to variational Bayesian inference, as in equations 6 and 7.

We fit the model using 10 starts with randomized initial parameter values, and with a range of cluster numbers less than or equal to the number of genes in the data set (in the case of the *DREAM4* data, 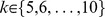) and then accept the model that has the highest estimated marginal likelihood. Accepting the model with the maximum marginal likelihood is simpler than combining all models based on their likelihoods, when in fact it is rare for a second, different model to have a likelihood close enough (i.e. a log likelihood within 3 or 4) to the best model for it to make a significant impact on the interaction rankings.

We are concerned primarily with the transition matrix 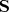 and the membership indicators 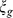; using posterior estimates for these, we can rank directed gene-gene interactions by their statistical strength. Specifically, for each directed cluster pair interaction 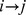 (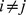), we calculate the posterior mean estimate for element 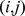 of 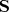 divided by its posterior standard deviation, assign this value to all possible directed pairs within the two clusters, and we rank by largest absolute value.

The *Octave* code implementing this algorithm–available at: http://code.google.com/p/bacon-for-genetic-networks-takes approximately 40 minutes on a single core of a 1.2 GHz processor for a single random start and a given number of clusters. Multiple starts and different numbers of clusters can be run in parallel; see the code for more details.

### Data

We used the *DREAM4 In Silico Network Challenge* data sets to evaluate the performance of our model. See [2], [20]–[22] for more details on the *DREAM* challenges. We utilized only the 10-gene time-series data, which consists of five simulated networks. For each of the networks, there are five time-series experiments, each with 20 time points. No simulated technical replicates were included, but random noise was added. The list of actual, “gold standard”, interactions was provided after the official challenges ended.

## Results

For each of the five data sets, each corresponding to a single gene regulatory network, we inferred the network using all available time-series (five each) and used the inferred interactions and the known gold standard to calculate the area under the receiver operating characteristic (AUROC) curve and the area under precision-recall (AUPR) curve, as in [1]. Table 1 gives the AUROC and AUPR for *BACON* both with and without clustering, as well as the corresponding scores from the *G1DBN* and *VBSSM* models, as reported in [1]. *BACON* gives an AUROC score better than both *G1DBN* and *VBSSM* in two out of five data sets– likewise for the AUPR scores–and is comparable to the other two algorithms in the remaining data sets. Given that *BACON* without clustering compares favorably with other algorithms, and that *BACON* with clustering gives the exact same results as *BACON* without clustering (the inferred number of clusters in each case was 10, the number of genes), we conclude that both versions of *BACON* give satisfactory results for these data sets.

**Table 1:** Algorithm results comparison for the DREAM4 networks.

	Algorithm	Data set 1	Data set 2	Data set 3	Data set 4	Data set 5
**AUROC**	*ACON*	**0.82**	**0.67**	0.72	0.81	0.88
	*BACON* (no clustering)	**0.82**	**0.67**	0.72	0.81	0.88
	*G1DBN*	0.73	0.64	0.68	**0.85**	**0.92**
	*VBSSM*	0.73	0.66	**0.77**	0.80	0.84
**AUPR**	*BACON*	**0.42**	0.36	**0.51**	0.49	0.57
	*BACON* (no clustering)	**0.42**	0.36	**0.51**	0.49	0.57
	*G1DBN*	0.37	0.34	0.45	**0.69**	**0.77**
	*VBSSM*	0.38	**0.41**	0.49	0.46	0.64

The area under the receiver operating characteristic (AUROC) curve and area under precision-recall (AUPR) curve for each of the five data sets. Here, we included *BACON* without clustering in order to establish that the plain DBN algorithm is generally as good as the other two DBN algorithms. The scores for *G1DBN* and *VBSSM* were taken from [7]. The best score for each data set is shown in bold.

However, the *DREAM4* time-series data are not typical; a single time-series with 20 time points is somewhat uncommon in practice (most experiments have 10 or fewer time points), and five independent time-series for the same gene network would be extremely rare. Thus, we subsequently consider each of the time-series individually, in order to see if an even more under-determined problem (only 20 data points for each of the 10 genes instead of 100) favors the model version with clsutering. We show in Table 2 the AUROC and AUPR of the 25 individual time-series (five from each of five data sets) for the *BACON* model both with and without clustering.

**Table 2:** Results of BACON on individual DREAM4 time series.

	Time-series	Data set 1	Data set 2	Data set 3	Data set 4	Data set 5
**AUROC**	1	0.68 (**0.71**)	0.61 (0.61)	0.64 (0.64)	0.62 (0.62)	0.57 (0.57
	2	**0.75** (0.66)	0.70 (0.70)	**0.68** (0.66)	0.77 (0.77)	0.64 (0.64)
	3	**0.67** (0.61)	0.62 (0.62)	**0.65** (0.60)	**0.60** (0.58)	0.65 (0.65)
	4	**0.61** (0.53)	0.66 (0.66)	0.59 (0.59)	**0.60** (0.60)	**0.74** (0.66)
	5	**0.66** (0.63)	0.64 (0.64)	0.59 (0.59)	0.76 (0.76)	0.78 (0.78)
**AUPR**	1	0.24 (**0.39**)	0.24 (0.24)	0.32 (0.32)	0.19 (0.19)	0.23 (0.23)
	2	**0.42** (0.27)	0.34 (0.34)	0.28 (**0.30**)	0.26 (0.26)	0.32 (0.32)
	3	**0.30** (0.19)	0.21 (0.21)	**0.24** (0.19)	0.15 (**0.16**)	0.24 (0.24)
	4	**0.24** (0.16)	0.38 (0.38)	0.21 (0.21)	**0.24** (0.18)	**0.27** (0.23)
	5	**0.22** (0.19)	0.20 (0.20)	0.17 (0.17)	0.34 (0.34)	0.33 (0.33)

For each of five individual time-series in each of the five data sets, the area under the receiver operating characteristic (AUROC) curve and area under precision-recall (AUPR) curve. For each time series, we give two of each score, one for *BACON* with clustering and one for *BACON* without clustering (in parentheses). The higher of the two scores appears in bold. If the two scores are identical, neither is in bold.

In many cases, the with-clustering and without-clustering scores were identical–i.e. 10 clusters is optimal–but in several other cases, fewer clusters gave a higher marginal likelihood score, and the corresponding AUROC and AUPR were indeed better, more often than not. Specifically, for 15 of the 25 time-series, *BACON* with clustering performed identically to the version without, but in seven cases, the version with clustering gave higher scores for both AUROC and AUPR. In only one case, the without-clustering version outperformed the with-clustering version in both AUROC and AUPR. These tallies are summarized in Table 3. Clearly, for smaller data sets such as a single time series, there is some benefit to be had from clustering the genes, when compared to non-clustering DBNs.

**Table 3:** Results comparison: with vs without clustering.

			Higher AUPR	
		with clustering	equal	without
	with clustering	7	0	2
**Higher AUROC**	equal	0	15	0
	without	0	0	1

Among the five individual time-series in each of the five data sets (25 total time series), here we give a tally of how many times *BACON* with clustering outperformed *BACON* without clustering, or vice versa, or if the AUROC and AUPR scores are equal.

## Discussion

Inferring gene regulatory networks from expression data is not usually easy, but it is common and often useful. Because of the under-determined nature of the problem–there are more parameters than data points–some reduction of the parameter set is often necessary in order to reach any meaningful conclusion at all. Sometimes, we can accomplish this through heuristic methods and decisions about which data are more important prior to the main statistical analysis. Other times, this is not desirable. In this paper, we present a probabilistic model of time-series gene expression with an integrated, theoretically sound method of parameter space reduction. We have described its implemetation and use, including a simple analytically-tractable example in which clustering is advantageous to network inference even if no “true” cluster exists, and if we are not at all concerned with cluster membership.

Many of the expectations we had for the Bayesian model turned out to be true. In particular, we expected the model to favor clustering mainly in data sets with few samples; in fact, the model preferred (via the likelihood function) not to cluster when we included all data for each network (100 samples, 20 from each of five time-series), but elected to cluster for 10 of the 25 separate time series (20 samples each). Likewise, because of the under-determined nature of network inference, we also expected the clustering model to perform better than a model without clustering if there are fewer samples. This also proved true; of the 10 time-series for which the model's marginal likelihood was highest for less than 10 clusters, seven were indeed better than without clustering (when comparing both AUROC and AUPR scores), and only one proved worse.

We believe that probabilistic clustering could be very useful in gene network inference, though there are disadvantages. For one, the computational time is generally much higher when clustering. This is due to the need to do model fits for a range of possible cluster numbers. For the purposes of this paper, in addition to doing the 10 random starts for the non-clustering model version, we do 10 random starts for the cluster quantities we wish to consider. Of course, the algorithm is much faster for smaller cluster numbers, as the size of the parameter of primary interest, the interaction/transition matrix, varies with the square of the number of clusters. It would likely be beneficial, in the case of very large data sets, to use a sequential or iterative search over the number of clusters, rather than use the exhaustive search method as we have here, but we leave that for a future publication.

In summary, we have shown that there are benefits to be had by clustering genes as part of a network inference algorithm. The potential for significant correlation among genes is high in typical time-series data sets, particularly those with few samples. The algorithm we have presented here, which we call *BAyesian Clustering Over Networks* (*BACON*), can help avoid the negative consequences of inter-gene correlation for the purposes of network inference. In our tests, the algorithm outperformed its non-clustering version in 7 out of 25 time-series from the *DREAM4 Challenge*, underperforming only once, and most often electing to disregard clusters when the data did not support it. Therefore, we feel that there are significant benefits of using probabilistic clustering to aid in the inference of gene regulatory networks.

Source code (*GNU Octave*), more information about the software for BAyesian Clustering Over Networks, (*BACON*) and sample data can be found at: http://code.google.com/p/bacon-for-genetic-networks.
