# Correction: Improved Inference of Gene Regulatory Networks through Integrated Bayesian Clustering and Dynamic Modeling of Time-Course Expression Data

**DOI:** 10.1371/annotation/64541b73-5ebc-4f5b-acab-241ecf2b3203

**Published:** 2014-01-02

**Authors:** Brian Godsey

An error occurred in the second row of the second column of Table 1. The acronym "ACON" should read "BACON."

Errors occurred in the references of this article in the Methods section. The reference numbers were replaced by question marks. In the first sentence of the fourth paragraph of the Methods section, the references should read "see [18] and [19]." In the second sentence of the fifth paragraph of that same section, the reference should read "as in [8]."

An error occurred in Equation 7 in which there is extra white space below the variables between the brackets.

